# Thrombotic Complications in Patients with COVID-19: Pathophysiological Mechanisms, Diagnosis, and Treatment

**DOI:** 10.1007/s10557-020-07084-9

**Published:** 2020-10-19

**Authors:** Aleksandra Gąsecka, Josip A. Borovac, Rui Azevedo Guerreiro, Michela Giustozzi, William Parker, Daniel Caldeira, Gemma Chiva-Blanch

**Affiliations:** 1 1st Chair and Department of Cardiology, Medical University of Warsaw, Warsaw, Poland; 2 Department of Pathophysiology, University of Split School of Medicine, Split, Croatia; 3 Cardiology Department, Hospital do Espírito Santo, Évora, Portugal; 4 Internal Vascular and Emergency Medicine and Stroke Unit, University of Perugia, Perugia, Italy; 5 Cardiovascular Research Unit, University of Sheffield, Sheffield, UK; 6 Centro Cardiovascular da Universidade de Lisboa (CCUL), Faculdade de Medicina, Univerisdade de Lisboa, Lisbon, Portugal; 7 Cardiology Department, Hospital Universitário de Santa Maria (CHULN), Avenida Professor Egas Moniz, 1649-028 Lisbon, Portugal; 8 Department of Endocrinology and Nutrition, August Pi i Sunyer Biomedical Research Institute (IDIBAPS), Hospital Clínic of Barcelona, Barcelona, Spain; 9 Spanish Biomedical Research Network in Physiopathology of Obesity and Nutrition (CIBEROBN), ISCIII, Madrid, Spain

**Keywords:** COVID-19, SARS-CoV-2, Thrombosis, Inflammation, Venous thromboembolism, Prophylaxis

## Abstract

**Introduction:**

Emerging evidence points to an association between severe clinical presentation of COVID-19 and increased risk of thromboembolism. One-third of patients hospitalized due to severe COVID-19 develops macrovascular thrombotic complications, including venous thromboembolism, myocardial injury/infarction and stroke. Concurrently, the autopsy series indicate multiorgan damage pattern consistent with microvascular injury.

**Prophylaxis, diagnosis and treatment:**

COVID-19 associated coagulopathy has distinct features, including markedly elevated D-dimers concentration with nearly normal activated partial thromboplastin time, prothrombin time and platelet count. The diagnosis may be challenging due to overlapping features between pulmonary embolism and severe COVID-19 disease, such as dyspnoea, high concentration of D-dimers, right ventricle with dysfunction or enlargement, and acute respiratory distress syndrome. Both macro- and microvascular complications are associated with an increased risk of in-hospital mortality. Therefore, early recognition of coagulation abnormalities among hospitalized COVID-19 patients are critical measures to identify patients with poor prognosis, guide antithrombotic prophylaxis or treatment, and improve patients’ clinical outcomes.

**Recommendations for clinicians:**

Most of the guidelines and consensus documents published on behalf of professional societies focused on thrombosis and hemostasis advocate the use of anticoagulants in all patients hospitalized with COVID-19, as well as 2-6 weeks post hospital discharge in the absence of contraindications. However, since there is no guidance for deciding the intensity and duration of anticoagulation, the decision-making process should be made in individual-case basis.

**Conclusions:**

Here, we review the mechanistic relationships between inflammation and thrombosis, discuss the macrovascular and microvascular complications and summarize the prophylaxis, diagnosis and treatment of thromboembolism in patients affected by COVID-19.

## Introduction

The severe acute respiratory syndrome coronavirus 2 (SARS-CoV-2), responsible for the coronavirus disease 2019 (COVID-19), originated in Wuhan, China, in December 2019. SARS-CoV-2 is a new ribonucleic acid (RNA) virus strain from the *Coronaviridae* family, firstly described by Zhou et al. in February 2020 [1]. In March 2020, COVID-19 was declared a pandemic by the World Health Organization (WHO), involving at the end of May more than 100 countries worldwide, number of cases and deaths rapidly increasing every day. On 25th June 2020, the WHO’s 157th Situation Report of COVID-19 announced that more than 9 million people worldwide had been infected by this virus, which has caused almost 480,000 deaths in barely 6 months. Europe and America have been the most affected regions, accounting for almost 80% of worldwide cases and deaths [2]. Despite SARS-CoV-2 strains having a within-sequence identity of 99.98%, it has been classified in 2 haplogroups (A and B), the first being the predominant worldwide and the second being more prevalent in America and Asia than in Africa and Europe [3]. Nevertheless, it remains unclear whether the severity and outcome of the disease are related to the virus strain.

SARS-CoV-2 fundamentally uses the angiotensin-converting enzyme 2 (ACE2) receptor to enter the cell through the binding with spike-like protein (S protein), although other cell receptors may be alternatively bound. In addition, ACE2 plays a major role in viral proliferation [4]. ACE2 is widely expressed in different organs and tissues, which concurs with the panoply of clinical systemic symptoms and multiorgan dysfunction presented with COVID-19, such as respiratory, renal, hepatic, gastrointestinal, and cardiac damage [5]. Both the down- and upregulation of ACE2 appear to play a major role in heart failure, hypertension, myocardial infarction, and overall cardiovascular disease (CVD) [6]. On those grounds, it is not surprising that patients with underlying CVD have 1.7-fold higher mortality rate caused by COVID-19 than patients with chronic respiratory disease, despite the respiratory system being the principal target of the virus [7].

Emerging evidence points to a strong association between severe clinical presentation of COVID-19 and increased risk of thromboembolism, although the mechanisms behind this are not completely understood [8]. Several risk factors have been described for such an association, such as systemic hyperinflammation driven by the coronavirus infection, hypoxia, and comorbidities associated with hospitalization of critical patients [9]. Whence, understanding the clinical and mechanistic features of COVID-19 severity and mortality is critical to identify and treat patients at the highest thromboembolic risk and mortality. We aim to review the mechanistic relationships between inflammation and thrombosis, discuss the macrovascular and microvascular complications, and summarize the diagnosis, prophylaxis, and treatment of thromboembolism in patients affected by COVID-19.

## Mechanistic Relationships Between Inflammation and Thrombosis

### Pathophysiology of Thrombosis

The thrombotic response involves activation of platelets and the coagulation cascade [10]. Remembering Virchow’s triad, changes in the blood flow, constituents, or vessel wall can precipitate thrombosis [11]. Platelet activation is initiated by several agonists, including collagen via glycoprotein (GP) VI receptors and thrombin via protease-activated receptors (PAR) 1 and 4. Upon activation, several key processes occur. First, arachidonic acid is converted to thromboxane A_2_, a potent pro-aggregatory and vasoconstrictive factor [12]. Second, platelets degranulate. Dense granules, containing adenosine diphosphate (ADP), fuse with the cell membrane [13]. ADP then acts on platelet P2Y_1_ and, most importantly, P2Y_12_ receptors, further stimulating and amplifying platelet activation [14]. Similarly, alpha granules, containing P-selectin as well as other proinflammatory and procoagulant factors, fuse with the membrane. P-selectin binds to a range on inflammatory cells, including neutrophils and monocytes [15]. Thirdly, through calcium mobilization and dephosphorylation of vasodilator-stimulated phosphoprotein (VASP), platelets undergo shape change from discoid to stellate forms, meaning physical aggregation occurs [16]. Conformational change in the GP IIb/IIIa receptor, which forms cross-links with other GP IIb/IIIa, strengthening platelet-platelet binding, and also stimulating other platelets to activate via outside-in signalling [17]. In addition, platelets and vascular and blood cell also release extracellular vesicles by outward blebbing, which are small particles released by almost all cell types when activated or injured, and composed by multiple bioactive molecules such as RNA, miRNA, cytokines, transcription and growth factors, and even small amounts of DNA, and lipids (they are rich in phospholipids) because they are enveloped in a lipid bilayer cell-derived membrane [18, 19]. Some extracellular vesicles expose phosphatidylserine in their external layer and therefore elicit a 50- to 100-fold higher procoagulant activity than activated platelets [20].

In addition to platelets and extracellular vesicles, activation of the coagulation cascade plays an important role in thrombosis [21]. Broadly divided into two converging pathways, activation of the extrinsic pathway by tissue factor and/or contact activation of the intrinsic pathway, coagulation cascade leads to activation of factor X, a constituent of the prothrombinase complex, resulting in thrombin generation. Thrombin cleaves soluble fibrinogen to insoluble fibrin, which forms interweaving strands, further stabilized by factor XIII. There is interplay between the coagulation cascade and platelets. Not only can thrombin activate platelets through PAR1 and PAR4, but also conversely platelets themselves can catalyze thrombin generation via membrane scramblase activity [22].

Although there is considerable overlap between platelet- and coagulation-mediated effects, the prominent mechanism of initiation varies by setting. Platelet activation is typically most prominent, whereas, [23] whereas in the venous circulation activation of the coagulation cascade predominates [24].

### The Inflammatory Response

The inflammatory response includes a complex network of factors, triggered by insults such as infection, trauma, or toxicity. Broadly, this is initiated by damage pattern recognition receptors found on a range of leukocytes [25]. This leads to recruitment and translocation of other leukocytes, release of cytokines and other inflammatory mediators, activation of complement, and physical attack of pathogens or infected cells [26]. Specifically in COVID-19-associated systemic inflammation, severe disease is associated with increased levels of cytokines such as interleukin (IL)-6, tumor necrosis factor (TNF)-α, and IL-2R [27]. Levels of, for example, IL-6 have also been associated with outcomes in severe COVID-19 [28].

### Role of Platelets During Inflammation

Platelets too play an important role in the regulation and enactment of the inflammatory response. Acute inflammation, for example, during endotoxemia, is associated with increased number of platelet-monocyte and platelet-neutrophil aggregates, mediated via enhanced P-selectin expression. Inhibiting platelets with P2Y_12_ inhibitors in this setting leads to in lower detectable plasma levels of cytokines such as IL-6 and TNF-α [29, 30].

Platelet reactivity, measured by numerous assays, is increased during acute inflammatory states, for example, sepsis [31]. Platelets themselves are acute phase reactants and inflammation induces thrombocytosis, mediated by an increase in thrombopoetin levels, potentiated by interleukin-6 [32]. Moreover, markers correlating closely with thrombotic risk, such as mean platelet volume and immature platelet fraction, or increased levels of circulating extracellular vesicles derived from platelets and leukocytes are increased during acute inflammation [33–35]. Raised circulating levels during inflammation of other platelet agonists such as adrenaline, via α_2_ receptors and serotonin (5-HT), via 5-HT_2A_ receptors, may also contribute to enhanced reactivity [36, 37].

### Role of Acellular Coagulation During Inflammation

Acute inflammation, triggered by a virus infection, for instance, is also associated with prothrombotic changes in fibrin clot dynamics, including increased fibrin strand density and clot turbidity [29]. Similarly, levels of markers such as D-dimer and fibrinogen may be elevated. Thrombin generation, which is increased during inflammation [38], drives not only coagulation and platelet activation but also inflammation more directly through promotion of leukocyte recruitment [39]. Conversely, severe inflammation can lead to such an increase in prothrombotic tendency that widespread microvascular thrombosis occurs, known as disseminated intravascular coagulation. This results in a fall in detectable clotting factors as these are consumed rapidly, and can paradoxically lead to reduced hemostatic function and therefore increased bleeding risk [40].

### Effect of Inflammation on the Endothelium

Endothelial function is adversely affected during systemic inflammation. In particular, the release of von Willebrand factor (vWF) is increased, which facilitates platelet-endothelium and platelet-platelet binding, On the contrary in the activity of the antithrombotic factors tissue factor pathway inhibitor and protein C are reduced[41]. In the arterial circulation, inflammation can also drive atheromatous plaque progression, impacting on local hemodynamics and also on plaque stability, making plaque rupture or erosion events, which can trigger thrombosis, more likely [42].

## Macrovascular Complications

One-third of patients hospitalized due to severe COVID-19 develop macrovascular thrombotic complications which are associated with an increased risk of in-hospital mortality [43, 44]. These complications include especially venous thromboembolism (VTE), and also stroke and acute myocardial infarction.

### Venous Thromboembolism

Elevated D-dimer concentration and thrombotic microangiopathy in pulmonary vessels on autopsy have raised the concern of pulmonary embolism (PE) as a reason of acute respiratory failure in patients with COVID-19. The in-hospital incidence of acute PE differs among studies, with the highest rate in severely ill patients admitted to intensive care unit (ICU) [45–56]. In a case series of 12 patients with severe COVID-19, post-mortem autopsies showed that VTE occurred in 7 of 12 (58%) patients, with PE being the direct cause of death in 4 of them (33%) [57]. To date, the largest published study included 388 patients [45]. Of these, 16% patients were admitted to the ICU. The cumulative rate of thromboembolic event was 21%, rising from 7% of patients admitted to the general ward to 28% of patients in the ICU. By pooling the data of all the studies (1765 patients) reporting the frequency of VTE in COVID-19, the overall incidence of VTE was 21.9% [8]. This cumulative incidence ranged from 31.3% in studies that included more than 75% ICU patients to 8.6% in studies that included less than 75% ICU patients. The high heterogeneity observed between the studies reflects the discrepancies of the inclusion criteria (e.g., patients with PE only vs. patients with PE with/without deep vein thrombosis [DVT]), thromboprophylaxis strategies, and definition of outcomes. This heterogeneity may lead to bias, highlighting the urgent need for further high-quality prospective research.

Only a few studies have reported the anatomical location of acute PE in COVID-19 patients [45, 47, 49, 58]. In a retrospective study of 137 patients with COVID-19, in which all patients underwent computed tomography pulmonary arteriography (CTPA) of the pulmonary arteries, a total of 32 PE were identified, of which ten were proximal PE, 18 involved segmental pulmonary arteries, and the remaining four PE were multiple subsegmental pulmonary arteries [58]. The segmental and subsegmental location of PE was also the most common in other studies [45, 49, 53]. It should be noted that only about one-third of COVID-19 patients undergo CTA during the hospital stay [45, 49, 53]. The risk of infection for both operators and other patients and the difficulties in performing CTPA in mechanical ventilated patients in prone position are the main reasons for the relatively low frequency of CTPA in these patients. Consequently, it cannot be excluded that the incidence of acute PE has been underestimated in patients with COVID-19.

Current data on the incidence of DVT in patients with COVID-19 are rather poor. In a recent study of 143 patients with COVID-19 undergoing ultrasonography of the lower limbs, DVT was found in 46.1% of patients [59]. Of these, 23 (34.8%) were proximal and 43 (65.2%) were distal DVTs. The Padua prediction score of 4 or higher and a D-dimer greater than 1.0 μg/ml were significantly associated with a more than 4-fold increased risk of DVT. Noteworthy, among distal vein thrombosis, 65% was in the intramuscular veins, regardless of whether it was symptomatic or asymptomatic. The clinical and prognostic meaning of asymptomatic distal DVT in these patients remains to be determined.

### Arterial Thrombosis

Both stroke and acute myocardial infarction have been described in patients with COVID-19. In observational studies, the proportion of COVID-19 patients with stroke ranges from 2.7 to 3.8% [45, 47, 48, 50, 53]. By pooling the available data (973 patients), the overall prevalence of in-hospital acute stroke is 3.5% (95% CI 2.4–4.8%), without statistical heterogeneity between the studies [8]. Both ischemic and hemorrhagic stroke can complicate the course of COVID-19. In a series of 6 patients, 4 patients had an ischemic stroke and 2 patients a hemorrhagic stroke [60]. Acute stroke in patients with COVID-19 is often associated with pre-existing cardiovascular risk factors and is a negative prognostic factor [61]. The mechanisms by which SARS-CoV-2 infection can trigger stroke depend on the associated pathogen and host characteristics. The presence of a prothrombotic state or a vasculitis-like mechanism may partly explain this association [60].

Myocardial injury indicated by increased troponin level may occur in 7–17% of COVID-19 patients admitted to the general ward and in 22–31% of those admitted to the ICU [44, 62, 63]. In a recent meta-analysis of 8 studies from China including 46,248 infected patients, 7% of patients experienced myocardial injury (22% of the critically ill), as evidenced by elevated cardiac troponin [64]. Noteworthy, patients with myocardial injury had higher in-hospital mortality (37.5%) than patients with cardiovascular disease (CVD) but without myocardial injury (13.3%), or patients without CVD (7.6%). Moreover, if myocardial injury was present in patients with pre-existing CVD, the mortality increased even more (69.4%) [65]. It was also demonstrated that myocardial injury is an equivalent to previous myocardial infarction in terms of mortality risk in COVID-19 patients [43]. Clearly, myocardial injury and underlying CVD markedly deteriorate the prognosis in COVID-19. The possible mechanisms explaining this association include (i) cytokine storm, (ii) microangiopathy, (iii) viral myocarditis, (iv) stress-induced cardiomyopathy, and (iv) classic myocardial infarction due to infection-induced atherosclerotic plaque instability [66, 67].

The management of both acute ischemic stroke and acute myocardial infarction can be challenging in these patients due to the need of urgent reperfusion and frequent lack of standard procedures to perform reperfusion in COVID-19 patients, essential to ensure the best care of patients. The role of veno-arterial extracorporeal membrane oxygenation (ECMO) as a form of rescue therapy in the case of COVID-19-associated cardiovascular collapse is currently under investigation.

## Microvascular Complications

Microvascular thrombosis is defined as pathological occlusion of microvessels (arterioles, capillaries, and venules) by platelet- and/or fibrin-rich thrombi [68]. Classically, microvascular thrombosis includes thrombotic microangiopathies (e.g., thrombocytic thrombocytopenic purpura, hemolytic-uremic syndrome) and disseminated intravascular coagulation (DIC) [69]. Microvascular thrombosis is a diagnostic challenge, because (i) microthrombi are difficult to visualize due to their small size (often ≤ 10 μm), (ii) microthrombi often occur only transiently, and (iii) specific biomarkers to detect them are lacking [68]. Clinically, microvessel occlusion leads to ischemia, with the effects ranging from alterations in plasma coagulation markers to severe multiorgan failure [70]. Although in most cases the evidence indicating a causal relationship between microvascular thrombosis and organ failure is difficult to obtain, microvascular thrombosis appears to have a critical importance in the course of COVID-19 [71].

Emerging evidence shows that severe COVID-19 can be complicated with coagulopathy of a prothrombotic character and is associated with poor prognosis [72]. Although many patients with severe COVID-19 present with coagulation abnormalities that mimic thrombotic microangiopathy or DIC, COVID-19-associated coagulopathy has distinct features [73]. The first autopsy series of four patients who died due to severe COVID-19 demonstrated a unique pathological picture, with the presence of diffuse microthrombosis and hemorrhage along with abundant intravascular megakaryocytes in all major organs, including the lungs, heart, kidneys, and liver [74]. Since the gross pulmonary thromboembolism and parenchymal inflammation were absent, this picture has been described as pauci-inflammatory thrombogenic vasculopathy. Another autopsy series of five patients with COVID-19 and acute respiratory distress syndrome (ARDS) demonstrated the lung and skin damage consistent with complement-mediated microvascular injury, whereas the hallmarks of classic ARDS with diffuse alveolar damage and hyaline membranes were not prominent [75]. The distinct respiratory distress syndrome accompanying severe COVID-19 has been termed microvascular COVID-19 lung vessels obstructive thromboinflammatory syndrome (MicroCLOTS) [76] or lung-restricted vascular immunopathology [77].

**Fig. 1:**
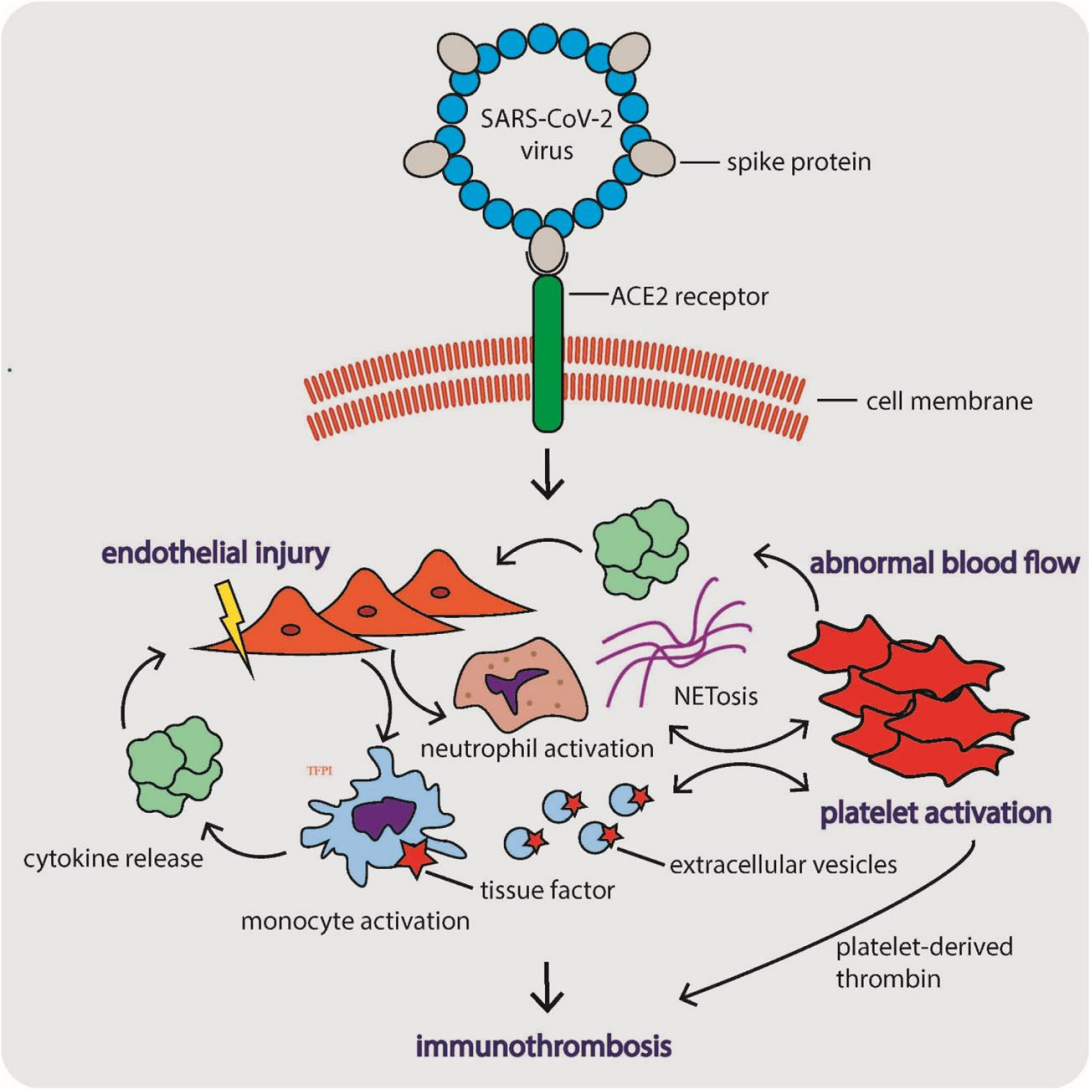
The pathophysiological mechanisms underlying COVID-19-associated coagulopathy. These mechanisms seem to follow Virchow’s triad, including (i) diffuse endothelial cell injury, (ii) abnormal blood flow dynamics, and (iii) uncontrolled platelet activation [71]

The pathophysiological mechanisms underlying COVID-19-associated coagulopathy are summarized in Fig. 1. These mechanisms seem to follow Virchow’s triad, including (i) diffuse endothelial cell injury, (ii) abnormal blood flow dynamics, and (iii) uncontrolled platelet activation [71]. SARS-CoV-2 enters the target cells through the ACE2 receptors, which are widely expressed on the surface of lung epithelial cells, and arterial and venous endothelial cells in multiple organs [78]. Virus infection causes direct endothelial injury, dysfunction, and prothrombotic gene expression [79]. In addition to the direct effect, the virus triggers innate immune responses, including the activation of monocytes and complement cascade, responsible for a massive local release of proinflammatory cytokines and aggravation of endothelial injury and microvascular thrombosis, termed immunothrombosis [76]. The major mediators of immunothrombosis include tissue factor (TF) and neutrophils. TF, exposed particularly by activated monocytes and monocyte-derived extracellular vesicles, is a major initiator of thrombosis in vivo [80]. Neutrophils, in turn, stabilize microthrombi via the release of neutrophil extracellular traps (NETs) and neutrophil elastase, which immobilize inflammatory cells and promote intravascular fibrin formation via degradation of TF antagonist, a tissue factor pathway inhibitor [81].

Abnormal flow dynamics results from high blood concentrations of vWF, cell-free DNA, histones, and viral RNA in course of COVID-19, which all cause factor XI activation, thrombin generation, and intravascular fibrin formation, leading to change in blood flow rheology [82]. Platelet activation is induced both indirectly by NETs and directly by virus infection. NETs lead to platelet activation via toll-like receptors on platelets and other cells, therefore integrating pulmonary infection, inflammation, and thrombosis [83, 84]. There is data showing that platelets and megakaryocytes may have receptors for viruses [85–87] and could be infected by earlier SARS-CoV [88]. Although currently there is no evidence of direct infection of platelets or megakaryocytes by SARS-CoV-2, the abundance of megakaryocytes in the lungs at autopsy seems to co-localize with platelet-rich thrombi [7]. Altogether, the immunothrombosis pathway in the lungs might be a starting point for the systemic propagation of COVID-19 infection, but more research is needed to elucidate this process.

Altogether, the mechanisms underlying microvascular thrombosis in course of COVID-19 may be related to diffuse vascular endothelial cell injury, hyperimmune reaction of the host, and maladaptive platelet aggregation. Clinician awareness of microvascular thrombosis during COVID-19 infection is crucial, since prompt recognition may be life-saving [89].

## Diagnosis, Prophylaxis, and Treatment of Thromboembolism

Thrombosis and thromboembolic complications in patients hospitalized with COVID-19 should be assessed by integrating physical examination findings, patient history, results of laboratory tests reflecting hemostasis, and/or results of focused imaging investigations. Patients who exhibit signs and symptoms of DVT and have risk factors for thrombosis (previous thrombotic events, active malignancy, or current use of hormonal therapy), unexplained and sudden deterioration in respiratory status, and unexplained hypotension and/or tachycardia should be further clinically evaluated to rule out thrombotic complications. In patients with high suspicion of thrombosis, most readily available imaging studies such as bilateral compression ultrasonography of the legs and/or bedside point-of-care cardiac ultrasonography should be used to detect proximal DVT, myocardial thrombus, clot-in-transit to the pulmonary trunk or right ventricular dilatation [92–95]. However, negative findings of these tests are insufficient to fully exclude thrombosis [92–95]. If the underlying etiology of persisting hemodynamic and respiratory compromise is not detected by less complex imaging modalities, a CTPA should be undertaken. On the other hand, a normal D-dimer test performed with high-sensitivity assays in this population reasonably rules out VTE, while positive D-dimer test does not confirm thrombosis, and therefore, a further imaging workup is warranted. Table 1

**Table 1:** The summary of studies reporting the frequency of VTE complications in COVID-19 patients

Studies	Study design	No. of pts	Setting	Male	Mean age	Median follow-up	Rates of PE during follow-up	Incidence of VTE	Use of thrombophophylaxis
Lodigiani et al. [45]	R Single center	388	ICU and general ward	58%	66	10 days	4.4%	21%	Enoxaparin or nadroparin. ICU, 100%; Ward, 75%. Regimen not specified
Poissy et al. [46]	R Single center	107	ICU	59.1%	57	6 days	20.6%	20.4%	20 out of the 22 PE patients were on prophylactic LMWH or UFH, but exact agents not specified.
Klok et al. [47, 48]	R Multicenter	184	ICU	76%	64	10 days	37%	57%	Varied by center.
Middeldorp et al. [49]	R Single center	198	ICU and general ward	66%	61	5 days	17%	15% at 7 days 34% at 14 days	ICU: nadroparin 2850 IU BID if weight < 100 kg, and 5700 IU BID if weight > 100 kg. Ward patients had half this dose
Helms et al. [50]	R Multicenter	150	ICU	81%	63	NR	18%	NR	LMWH (exact agent not specified) 4000 Units per day or UFH 5–8 U/kg/h
Llitjos et al. [51]	R Double center	26	ICU	77%	68	NR	23%	NR	LMWH and UFH were used (exact agents not specified)
Thomas et al. [53]	R Single center	63	ICU and general ward	69%	59	8 days	9%	27%	All patients assessed for use of prophylaxis with weight-adjusted dalteparin
Leonard-Lorant et al. [54]	R Double center	106	ICU and general ward	66%	63.5	NR	30%	NR	Anticoagulant not specified. In PE + group, 78% were on prophylactic doses and 6% were on therapeutic doses.
Grillet et al. [55]	R Single center	100	ICU and general ward	70%	66	NR	23%	NR	NR
Bompard et al. [58]	R Double center	135	ICU and general ward	70%	64	5 days	23.7%	50% ICU 18% GW	All patients received standard dose of prophylaxis (Enox 4000 daily in GW, twice daily in obese and ICU patients)
Galeano-Valle et al. [90]	P Single center	24	General ward	58%	64.3	14 days	6.5%	NR	All patients received standard dose of prophylaxis (enoxaparin 4000 UI daily or bemiparin 3500 UI daily)
Hippensteel et al. [91]	R Single center	91	ICU	58%	55	NR	5.5%	26.1%	54.3% of patients received therapeutic anticoagulation

*P*, prospective; *R*, retrospective; *ICU*, intensive care unit; *NR*, not reported; *LMWH*, low molecular weight heparin; *UFH*, unfractionated heparin

Importantly, elevations of laboratory parameters indicating hypercoagulability at the admission of patients with COVID-19 are associated with an increased incidence of in-hospital thromboembolic events and mortality [44, 52, 72, 96–98]. For example, in a recent report including 2377 consecutive adults hospitalized with COVID-19, 76% of patients had elevated D-dimer at presentation which had prognostic value for the severe course of COVID-19 infection, including 2-fold higher odds for critical illness, thrombotic events, acute kidney injury, and death. Importantly, the rates of adverse events increased with the magnitude of D-dimer elevation [99]. The initial prothrombotic cascade in patients with COVID-19 is similar as in critical patients with DIC and consumptive coagulopathy; however, there are clear differences between COVID-19-associated coagulopathy and patients that have classical DIC. Notably, coagulopathy in COVID-19 patients is primarily characterized by the marked elevations in D-dimer and fibrin/fibrinogen degradation product levels while thrombocytopenia and prolongations of activated partial thromboplastin time (aPTT) and/or prothrombin time (PT) are less common or usually mild [100]. Accordingly, a hyperfibrinolytic consumptive DIC with a bleeding diathesis is a rare and unlikely event in these patients [100]. A worsening trend of coagulation parameters during an ICU stay generally indicates impending clinical deterioration, development of cytokine storm, and progression to multiorgan failure [101]. The procoagulant pattern in severe COVID-19 patients, assessed by standard laboratory and viscoelastic tests, revealed increased clot strength (CS), increased platelet and fibrinogen contribution to CS, elevated D-dimer levels, and hyperfibrinogenemia [102]. Similarly, a complete lack of fibrinolysis of a clot at 30 min and exuberant increase in D-dimer levels were strongly associated with thromboembolic events and renal failure [97]. Furthermore, levels of C-reactive protein (CRP), IL-6, factor VIII, vWF, and plasma viscosity are increased in these patients while antithrombin is modestly decreased thus clearly showing a cross-link between infection-induced inflammatory response and thrombosis, known as infection-induced thromboinflammation [100, 102–104]. A summary of the most important laboratory parameters reflecting hemostasis derangement in patients with COVID-19 is shown in Table 2.

**Table 2:** Laboratory tests reflecting hemostasis in patients with COVID-19 associated coagulopathy

Laboratory variable		COMMENTS
**Standard coagulation and platelet panel**		
D-dimer	**↑↑↑**	**Markedly elevated** 3- to 4-fold elevation associated with high mortality
FDPs	**↑↑**	**Elevated**
Fibrinogen	**↑↑** (↓)	**Elevated** Decreasing trend if patient’s condition progresses towards consumptive coagulopathy phenotype (e.g., DIC)
aPTT	**←→** (**↑**)	**In normal range** OR **slightly prolonged**
PT	**←→** (**↑**)	**In normal range** OR **slightly prolonged**
Platelet count	**←→** (**↓**) (**↑**)	**Near normal OR mildly decreased** Ranging from 100–150 × 10^9^ cells/L in 70–95% patients with severe COVID-19, platelet count < 100 × 10^9^ cell/L was detected in about 5% of severe COVID-19 patients. Could be slightly increased based on limited data from small cohorts
**Advanced rheological parameters**		
Plasma viscosity	**↑↑**	**Increased 2-fold**, on average
Factor VIII activity	**↑**	**Increased**
von Willebrand factor	**↑**	**Increased**
Antithrombin activitiy	(**↓**)	**Modestly decreased**
Free protein S	(**↓**)	**Modestly decreased**
Protein C	(**↑**)	**Modestly increased**

*aPTT*, activated partial thromboplastin time; *FDPs*, fibrin degradation products; *PT*, prothrombin time. **←→** indicates normal range. (**↑**) and (**↓**) denote modest increase or decrease, respectively; **↑** and **↓**, slightly increase or decrease, respectively; **↓↓** and **↓↓**, considerably increase or decrease, respectively; and **↑↑↑** and **↓↓↓** strongly increase or decrease, respectively

In terms of laboratory workup, measurements of D-dimer levels, PT/aPTT, thrombocyte count, and fibrinogen levels (if assays are available) are advised to identify hospitalized COVID-19 patients at a high risk of poor in-hospital outcomes, or to triage patients that would require hospital admission and close monitoring [105, 106]. Repeated assessments (every 2–3 days) of D-dimer, PT, and the platelet count at the minimum should be integrated into the diagnostic approach, as recommended by the documents of relevant societies [73, 106]. PT expressed as an international normalized ratio (INR) should not be used to monitor hemostasis in COVID-19 patients, since it mostly lies in the normal range. Early recognition and vigilant monitoring of coagulation abnormalities among hospitalized COVID-19 patients are critical measures to (i) identify patients at a high risk of poor prognosis, (ii) guide antithrombotic prophylaxis or treatment to prevent thromboembolic complications, and (iii) improve patients’ clinical outcomes.

Most of the guidelines and consensus documents published on behalf of professional societies focused on thrombosis and hemostasis advocate the use of prophylactic doses of anticoagulants in all patients hospitalized with COVID-19 in the absence of contraindications [105–108]. Likewise, extended-duration thromboprophylaxis with low molecular weight heparin (LMWH) or a non-vitamin K antagonist oral anticoagulants (NOAC) for 2 to 6 weeks post-hospital discharge might be applied to hospitalized COVID-19 patients, especially those with no bleeding risk factors and with high VTE risk post-discharge (most commonly defined as advanced age, long stay in the ICU, malignancy, prior VTE history, thrombophilia, severe immobility, D-dimer levels > 2 times of upper reference range, an IMPROVE VTE score ≥ 4, and similar). Such recommendations are made due to observed hypercoagulability and a robust number of studies that consistently showed a high incidence of thromboembolic complications, especially venous thromboembolism in hospitalized and particularly ICU-treated COVID-19 patients [45, 46, 48, 49, 52]. Likewise, most of the available documents support the possibility to initiate intermediate-dose LMWH in selected severe COVID-19 patients by individually balancing thrombotic and bleeding risk. Finally, in hospitalized COVID-19 patients with verified VTE, the aforementioned consensus statements stimulate the use of full therapeutic doses of antithrombotic drugs (LWMH as most preferred, followed by unfractionated heparin) in all patients without contraindications, with carefully monitoring renal function, thrombocyte count, and concomitant medications. Antithrombotic prophylactic and therapeutic regimens in various clinical scenarios as recommended by relevant societies are shown in Table 3.

**Table 3:** Antithrombotic prophylaxis and treatment regimens in various settings of COVID-19 infection according to international societies

**Society/Document** (*PubMed ID number*)	**Antithrombotic agent and dosage**	**Population**	**Duration of treatment**
**COVID-19 patients WITHOUT diagnosis of VTE**			
**International Society of Thrombosis and Haemostasis (ISTH)** (*PMID: 32338827*)	**LMWH** In standard VTE prophylactic dose, in the absence of contraindications*	**All COVID-19 patients who require hospitalization** (including non-critically ill patients)	**During the whole duration of hospital stay**
**STH – Subcommittee of Perioperative and Critical Thrombosis and Haemostasis of the Scientific and Standardization Committee** (*PMID: 32459046*)	**LMWH** (preferred agent, once daily) OR **UFH** (twice or thrice daily) OR **DOACs** (least preferred, due to interference w/ immunosuppressant and antiviral drugs), in the absence of relevant contraindications* All in standard VTE prophylactic doses** *Intermediate-dose LMWH in-hospital may be considered in severe patients*	**All non-ICU hospitalized COVID-19 patients**	**During the whole duration of hospital stay** Extended duration thromboprophylaxis with LMWH or DOAC for 2–6 weeks (14 days at least, up to 30 days) post-discharge in selected patients with low risk for bleeding and key VTE risk factors could be used****
	**LMWH** (preferred agent, once daily) OR **UFH** (twice or thrice daily) All in standard VTE prophylactic doses* *Intermediate-dose LMWH in-hospital can be considered in high-risk patients* Full-dose heparin treatment is not recommended	**All ICU hospitalized COVID-19 patients**	**During the whole duration of hospital stay** Extended duration thromboprophylaxis with LMWH or DOAC for 2–6 weeks (14 days at least, up to 30 days) post-discharge in selected patients with low risk for bleeding and key VTE risk factors could be used****
**Italian Society on Thrombosis and Haemostasis (SISET)** (*PMID: 32281926*)	**LMWH** OR **UFH** OR **fondaparinux** All in standard VTE prophylactic dose, in the absence of contraindications* *Intermediate-dose LMWH in-hospital (enoxaparin 4000 IU subcutaneously, twice daily) could be considered on an individual basis in patients with multiple risk factors for VTE* Full-dose heparin treatment is not recommended	**All hospitalized COVID-19 patients**	**During the whole duration of the hospital stay** Maintained at home for 7–14 days post-discharge in case of pre-existing or persisting VTE risk factors
**ISTH, North American Thrombosis Forum (NATF), European Society of Vascular Medicine (ESVM), International Union of Angiology (IUA), European Society of Cardiology (ESC) Working Group on the Pulmonary Circulation and Right Ventricular Function** *(PMID: 32311448)*	**LMWH**, once daily, preferred agent OR **UFH**, twice daily Both in standard VTE prophylactic dose, in the absence of contraindications* Mechanical VTE prophylaxis in patients with contraindications in immobilized patients Prophylactic anticoagulation is the only fully recommended modality *Intermediate-dose LMWH in-hospital could be an option in select patients (minority of the panel support this option)* Full-dose heparin treatment is not recommended	**Hospitalized patients with COVID-19** who have: respiratory failure or comorbidities such as active malignancy or heart failure, are bedridden or requiring intensive care	**During the whole duration of hospital stay** Extended duration thromboprophylaxis with LMWH or DOAC for up to 45 days among patients with high VTE risk and low risk of bleeding****
**COVID-19 patients WITH confirmed diagnosis of VTE**			
**International Society of Thrombosis and Haemostasis (ISTH)** (*PMID: 32338827*)	**Not discussed**	**N/A**	**N/A**
**ISTH – Subcommittee of Perioperative and Critical Thrombosis and Haemostasis of the Scientific and Standardization Committee** (*PMID: 32459046*)	**LMWH** (inpatient setting; a change from treatment-dose DOACs or VKAs to in-hospital LMWH should be considered in critical care patients wtih relevant concomitant medications, based on renal function and thrombocyte count) **DOACs** (post-hospital discharge setting) Standard VTE therapeutic doses in the absence of contraindications* *Prophylactic or intermediate-dose to treatment-dose regimen might be considered in patients without verified VTE but with worsening pulmonary status or ARDS*	**All hospitalized COVID-19 patients** **with established VTE**	**Duration of anticoagulation treatment should be at least 3 months**
**Italian Society on Thrombosis and Haemostasis (SISET)** (*PMID: 32281926*)	**LMWH** OR **UFH** OR **DOACs** Standard VTE therapeutic doses in the absence of contraindications*	**All hospitalized COVID-19 patients with established VTE**	**Duration of treatment in this scenario should be according to established classic guidelines for therapeutic anticoagulation of established VTE** In patients requiring therapeutic doses of LMWH or DOACs, a careful monitoring of renal function with anti-factor Xa or plasma DOAC levels assays should be instituted VKAs and DOACS significantly interfere with concomitant antiviral treatment and individualized risk/benefit approach should be applied for every patient
**ISTH, North American Thrombosis Forum (NATF), European Society of Vascular Medicine (ESVM), International Union of Angiology (IUA), European Society of Cardiology (ESC) Working Group on the Pulmonary Circulation and Right Ventricular Function** *(PMID: 32311448)*	**UFH**, parenteral (preferred in critical patients that might undergo procedures) OR **LMWH**, subcutaneous (preferred in patients unlikely to undergo procedures) **DOACs** OR **LMWH** (preferred as post-discharge therapy due to reduced need for contact with healthcare workers required for INR monitoring of VKAs) All in standard VTE therapeutic doses, in the absence of contraindications* Mechanical VTE prophylaxis in patients with contraindications in immobilized patients	**Hospitalized patients with COVID-19 with established VTE**	**Duration of treatment in this scenario should be according to established classic guidelines for therapeutic anticoagulation of established VTE**

*ARDS*, acute respiratory distress syndrome; *DOACs*, direct oral anticoagulants; *INR*, international normalized ratio; *LMWH*, low molecular weight heparin; UFH-unfractionated heparin; *VKA*, vitamin K antagonist; *VTE*, venous thromboembolism

*Active bleeding, thrombocyte count < 25 × 10^9^/L, severe renal impairment (close monitoring required)

**Treatment should be modified individually according to patient’s body weight, severity of thrombocytopenia (25–50 × 10^9^ cells/L) or worsening renal function; intermittent pneumatic compression devices might be used in patients in whom anticoagulant therapy is contraindicated

***Obese patients (body mass index ≥ 30) should be considered for the 50% increase in the dose of thromboprophylaxis; multimodal prophylaxis with mechanical methods such as intermittent pneumonic compression devices should be considered

****High-risk factors defined as advanced age, stay in the ICU, cancer, a prior history of VTE, thrombophilia, severe immobility, elevated D-dimer levels (> 2 times of upper normal range), and an IMPROVE VTE score of 4 or more

A treatment rationale for recommendations elaborated above is also partially based on the reported survival benefits, if the anticoagulation regimen is initiated in these patients. Of note, one study showed that treatment with prophylactic doses of LMWH was associated with lower 28-day mortality among patients with severe COVID-19 that had sepsis-induced coagulopathy score ≥ 4 or D-dimers > 6-fold of an upper limit of normal, compared with LMWH non-users [109]. However, the crude difference between the unselected group of patients that received LMWH vs. non-receivers in 28-day mortality endpoint was not significantly different. Similarly, another study found the significantly increased probability of survival in 2773 hospitalized COVID-19 patients if systemic anticoagulation was initiated, compared with if it was not, especially among patients that required mechanical ventilation [110]. One of the putative explanations for the beneficial effect of heparin and its derivatives in COVID-19 is based on the fact that heparins exhibit pleiotropic actions beyond anticoagulation, including direct anti-inflammatory action, as demonstrated by the decreased levels of inflammatory biomarkers in pre-clinical and clinical settings [111, 112]. Given that the severe inflammatory response in COVID-19 is the principal driver of coagulopathy, the concept of using heparin and its derivatives seems clinically validated as they likely blunt both thrombotic and inflammation pathways, thus improving outcomes in these patients.

## Recommendations for Clinicians

COVID-19 caused by SARS-CoV-2, at the severest stages might be considered as a thromboinflammatory disease rather than an infection. In fact, the presence of microthrombotic pulmonary disease led to the suspicion that pulmonary macro- or microembolism could play a role in the pathophysiology and clinical deterioration of COVID-19 patients [1, 113]. The diagnosis of PE or MicroCLOTS may be challenging, because there are some overlapping features between pulmonary embolism and severe COVID-19 disease, such as dyspnoea, high levels of D-dimers, right ventricle with systolic dysfunction or enlargement, and ARDS. Most of the inpatients with COVID-19 should receive at least prophylactic anticoagulation in the absence of contraindication, particularly those at ICU, due to high reported incidence of VTE in this context [8, 89]. There are some observational studies suggesting that heparin improves the disease prognosis when the indication bias would suggest a worse prognosis (i.e., patients that received anticoagulation probably had an indication that could worsen the prognosis but the data suggests otherwise) [89, 109]. As there is no guidance for deciding the intensity of anticoagulation (prophylactic, intermediate, or therapeutic), the decision-making process should be made in individual-case basis, while studies are ongoing (NCT04377997, NCT04362085, NCT04367831). The suspicion of PE should be based in clinical grounds (unexplained chest pain, unexplained RV dysfunction, unilateral lower limb swelling) and not only in biomarkers such as D-dimers. It is known that D-dimers are frequently high in COVID-19 inpatients [62], they are a prognosis biomarker [114], but it is not clear if they reflect the existence of macrovascular thrombosis and/or the need to screen systematically VTE in these patients [108].

As for myocardial injury/infarction, there are also challenges because elevated troponin levels are often seen in COVID-19 patients, and the cause of troponin release may be multifactorial. First, both plaque rupture can occur during severe COVID-19 (type 1 myocardial infarction), and other causes of troponin release can be present, including imbalance between blood/oxygen supply and myocardial requirements, SARS-CoV-2 myocarditis, Tako-tsubo cardiomyopathy, or right heart failure (type 2 myocardial infarction). Patients with ST-elevation myocardial infarction (STEMI) or high-risk non-ST-elevation acute coronary syndrome (NSTE-ACS) should go to the catheterization laboratory, preferably the one dedicated to COVID-19, and followed by an ICU isolation bed [115]. For patients with COVID-19 and STEMI in a non-primary PCI center, fibrinolysis can be considered depending on the current timings for patient transfer for a primary PCI center [115]. In the remaining patients with COVID-19 and NSTE-ACS, physicians can opt out for optimal medical treatment alone for clinical stabilization while further testing, or coronary angiography should be performed after the infection is resolved [115].

Stroke in COVID-19 patients was not as common as VTE [8]. The approach to stroke is the same as non-COVID-19 patients, pursuing the vessel recanalization when indicated and when resources are available [116]. Ideally, these patients should be at COVID-19 stroke units, but logistic difficulties have been seen during the COVID-19 season [117]. In patients with severe COVID-19 disease, the development of stroke signs and symptoms should also raise the suspicion of paradoxical embolism through atrial septum defect.

In summary, COVID-19 is an infectious disease that leads to a proinflammatory and prothrombotic state, resulting in both micro- and macrovascular thrombosis, and both arterial and venous thrombotic events. Therefore, the early recognition and vigilant monitoring of the thrombotic complication of COVID-19 may be life-saving.

## Data Availability

Not applicable.
